# A Self-supported Graphene/Carbon Nanotube Hollow Fiber for Integrated Energy Conversion and Storage

**DOI:** 10.1007/s40820-020-0390-x

**Published:** 2020-02-25

**Authors:** Kai Liu, Zilin Chen, Tian Lv, Yao Yao, Ning Li, Huili Li, Tao Chen

**Affiliations:** Shanghai Key Lab of Chemical Assessment and Sustainability, School of Chemical Science and Engineering, and Institute of Advanced Study, Tongji University, Shanghai, 200092 People’s Republic of China

**Keywords:** Carbon nanotube, Graphene, Integrated, Energy conversion, Energy storage

## Abstract

Wearable fiber-shaped integrated energy conversion and storage devices have attracted increasing attention, but it remains a big challenge to achieve a common fiber electrode for both energy conversion and storage with high performance. Here, we grow aligned carbon nanotubes (CNTs) array on continuous graphene (G) tube, and their seamlessly connected structure provides the obtained G/CNTs composite fiber with a unique self-supported hollow structure. Taking advantage of the hollow structure, other active materials (e.g., polyaniline, PANI) could be easily functionalized on both inner and outer surfaces of the tube, and the obtained G/CNTs/PANI composite hollow fibers achieve a high mass loading (90%) of PANI. The G/CNTs/PANI composite hollow fibers can not only be used for high-performance fiber-shaped supercapacitor with large specific capacitance of 472 mF cm^−2^, but also can replace platinum wire to build fiber-shaped dye-sensitized solar cell (DSSC) with a high power conversion efficiency of 4.20%. As desired, the integrated device of DSSC and supercapacitor with the G/CNTs/PANI composite hollow fiber used as the common electrode exhibits a total power conversion and storage efficiency as high as 2.1%. Furthermore, the self-supported G/CNTs hollow fiber could be further functionalized with other active materials for building other flexible and wearable electronics.

**Electronic supplementary material:**

The online version of this article (10.1007/s40820-020-0390-x) contains supplementary material, which is available to authorized users.

## Highlights

- A novel self-supported hollow fiber with CNT array grown from graphene layer is designed and developed. The fiber provides a perfect conductive platform to deposit PANI on both inner and outer surfaces of the fiber with high mass loading.
- With G/CNTs/PANI hollow fiber as a common electrode, an integrated energy device with a total power conversion and storage efficiency of 2.1% is realized.

**Electronic supplementary material:**

The online version of this article (10.1007/s40820-020-0390-x) contains supplementary material, which is available to authorized users.

## Graphical abstract

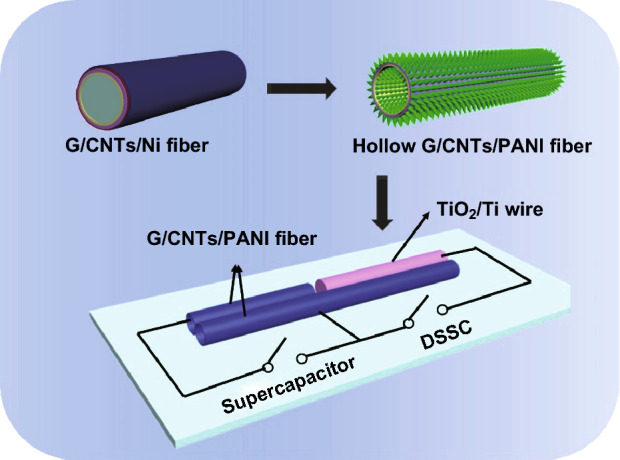

## Introduction

Due to their unique structure, fiber-shaped electronics not only show advantages of excellent flexibility and lightweight, but also can be easily woven or integrated into any other textiles, attracting great attentions from various fields, such as sensors [1–5], intelligent skins [6, 7], and biomedicine [8–10]. To power these fiber-shaped electronics with a good match, it is required to develop fiber-shaped wearable energy conversion and/or storage devices [11–13]. As a promising energy conversion device, solar cells that can convert solar energy into electricity have been widely studied, and fiber-shaped devices have been achieved [14, 15]. On the other hand, electrochemical supercapacitors with high power density and long cycling life that could be fabricated in fiber-like format through simple and environment-friendly approaches have been intensely investigated so far [16–18]. For both fiber-shaped solar cells and supercapacitors, high conducting fiber electrodes are one of the most important parts in these fiber-shaped devices and play an important role in their performance. However, it remains a big challenge to achieve a common fiber electrode for building of integrated energy conversion and storage devices with high performance.

Benefiting from their large specific area, excellent mechanical, electrical, and electrochemical properties, carbon nanotubes (CNTs) and graphene have been widely used as efficient electrode materials for energy relative devices containing solar cells and supercapacitors [19–23]. In addition, CNTs and graphene could be easily assembled into fiber materials [24–26], which showed high mechanical and electrical conductivity. Compared with traditional electrodes of metal wires and conducting materials-coated polymer fibers [27, 28], fiber derived from CNTs and graphene exhibited more flexible and stable properties, showing great potential to be used as fiber electrode for fiber-shaped electronics. To date, fiber-shaped solar cells and supercapacitors have been developed by using fiber electrodes assembled from CNTs and/or graphene, and great achievements have been received [26, 29, 30]. To achieve high-performance devices, other active materials were often needed to be combined with CNTs or graphene fibers [31, 32]. However, only external surface of the fibers could be used for functionalization, which often resulted in composite fibers with low loading of active materials, greatly limited the performance of the fiber-shaped devices. High loading of active materials in fiber electrodes and high-performance fiber-shaped devices may be achieved if a hollow electrode was applied. Unfortunately, there were few efficient strategies to fabricate CNTs or graphene hollow fibers so far, because serious aggregation often occurred during their preparing process.

Here, we designed and developed a type of self-supported hollow G/CNTs fiber by covalently grown CNTs array outside of continuous graphene tubes. Through this design, the outer CNTs can well support the inner graphene tube and prevent aggregation of graphene when the substrate was removed. In addition, the synergistic effect between CNTs and graphene with seamless connection provides the obtained hollow G/CNTs fibers with excellent electrical and electrochemical properties, which enable them to be served as promising candidate electrodes for electronics. On the other hand, the unique hollow structure of G/CNTs fibers can be well maintained even they were immersed in solution, which can provide both inner space and outside surface for desired functionalization with high loading content. In this regard, we have successfully deposited aligned conductive polymer of polyaniline (PANI) on both inner and outer surface of hollow G/CNTs fibers by one-step in situ polymerization. The newly developed hollow G/CNTs/PANI composite fibers can not only be used as counter electrode to build fiber-shaped dye-sensitized solar cell (DSSC) with a high power conversion efficiency of 4.20%, but also be used for high-performance fiber-shaped supercapacitor (472 mF cm^−2^). The results indicated that the hollow G/CNTs/PANI composite fibers could be used as the common electrode to construct high-performance integrated energy devices. As desired, the developed fiber-shaped integrated device containing a DSSC and a supercapacitor exhibited a high overall photoelectric conversion and storage efficiency of 2.1%.

## Experimental Section

### Fabrication of G/CNTs Hollow Fibers

Multilayer graphene was grown around nickel (Ni) wire (diameter of 0.05 mm), which was alternately washed with the acetone and deionized water for several times before usage. Typically, the Ni wire was placed in a tubular furnace and heated from 25 to 1,000 °C with a mixture carrier gas consisting of argon (Ar, 400 sccm) and hydrogen (H_2_, 80 sccm). After the furnace maintaining at 1,000 °C for 10 min, methane (60 sccm) was introduced to grow graphene for 10 min, and the Ni wire grown with graphene (G/Ni) was put out after the temperature of furnace decreased to room temperature. Then, a catalyst of iron layer (1.5 nm) and a buffer layer of aluminum oxide (10 nm) were sequentially deposited on G/Ni wire by an electron-beam evaporation. Finally, the catalyst covered G/Ni wire was put into the tubular furnace for growth of CNTs with the mixture of Ar (200 sccm) and H_2_ (45 sccm) as carrier gas. After the sample was annealed in the furnace at 750 °C for 10 min, ethylene (5 sccm) was introduced to grow CNTs for 4 min. The height of the as-grown CNTs arrays depended on growth time. The substrate of Ni wire was removed by immersing the obtained samples in a mixed aqueous solution consisting of 20 mL concentrated nitric acid and 60 mL ferric chloride solution (3 mol L^−1^) for 24 h at the room temperature, and the obtained G/CNTs hollow fiber was washed with deionized water for several times to remove the residual metal salt.

### Preparation of G/CNTs/PANI Composite Hollow Fibers

The G/CNTs/PANI composite hollow fibers were obtained by in situ polymerization of aniline. In detail, ammonium persulfate (APS, 6.7 mM) was added into 15 mL aqueous solution consisting of 10 mM aniline and 1 M HClO_4_ and stirred in ice bath. Then, G/CNTs hollow fibers were immersed into the above-mentioned solution at 3 °C for 24 h. G/CNTs/PANI composite hollow fibers were obtained after being washed with deionized water.

### Fabrication of Two-electrode-based Fiber Supercapacitors

The polyvinyl alcohol (PVA)/H_3_PO_4_ gel electrolyte was prepared according to the previous literature [33]. One fiber electrode was placed on an elastic substrate and drop-coated with the polymer gel electrolyte, placed in a vacuum oven for 15 min. Then, another fiber electrode was placed overlapped onto the former electrode and drop-coated with PVA/H_3_PO_4_ gel electrolyte again. Finally, the ends of these two fiber electrodes were connected with copper wires for measurement.

### Fabrication of the Fiber-shaped Dye-sensitized Solar Cell

A titanium wire (Ti, diameter of 127 μm) was used to grow aligned titania (TiO_2_) nanotubes by electrochemical anodization at 60 V for 2 h. The electrolyte solution for electrochemical anodization was fabricated by dissolving of 2.63 mL H_2_O and 0.146 g NH_4_F in 50 mL glycol. A Ti wire and a piece of Pt plate were used as working electrode and the counter electrode, respectively. The anodized Ti wire was washed with deionized water and annealed in air at 500 °C for 1 h; after the temperature down to 120 °C, the annealed Ti wire was immersed into a solution of N719 (7.13 mg) in 10 mL mixed solvent of acetonitrile and tert-butyl alcohol (volume ratio of 1:1) to absorb dye molecules for 16 h. The DSSC was fabricated by parallelly ranging a dye-sensitized Ti wire and a G/CNTs/PANI hollow fiber together on an elastic substrate, followed by drop-coating electrolyte. The ends of these two fiber electrodes were connected with copper wires for measurement.

### Characterizations

The morphology and structure of materials were characterized by the field emission scanning electron microscopy (FESEM, Hitachi S-4800, operated at 5 kV) and high-resolution transmission electron microscopy (HRTEM, JEOL-2010 operated at 200 kV). The Raman spectra were measured by Raman spectrometer (Renishaw) equipped with a 514-nm laser. The electrochemical performance of supercapacitors was conducted with an electrochemical working station (CHI 760E, Chenhua, Shanghai). The mechanical properties of fiber materials were tested in a universal mechanical test machine (HY-0350, Shanghai Hengyi Co. Ltd). The current–voltage curves of solar cell were measured by a Keithley Model 2400 under a light intensity of 100 mW cm^−2^ (calibrated by a standard silicon solar cell) with a solar simulator (Oriel Sol3A). The water contact angles were performed with a contact angle measuring device (SDC-350). The weights of G/CNTs and G/CNTs/PANI fibers were measured by using high-precision electronic balance with the accuracy of 0.01 mg (BT25S, Sartorius).

## Results and Discussion

**Fig. 1:**
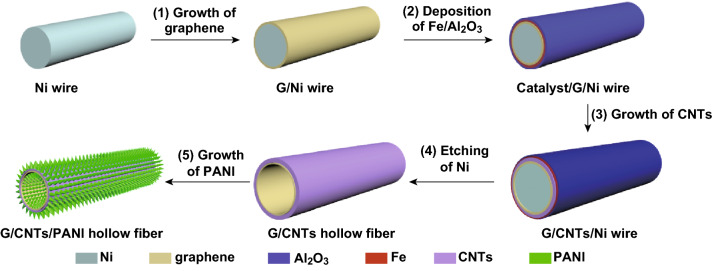
Schematic of the fabrication process of self-supported G/CNTs hollow fiber and its composite

**Fig. 2:**
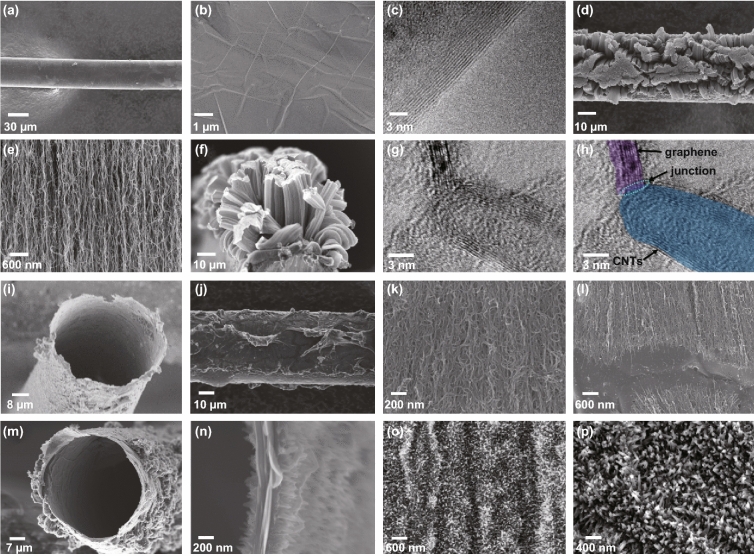
Structure of G/CNTs hollow fiber and its composite fiber. **a, b** SEM images of the as-grown graphene on a Ni wire. **c** TEM image of graphene with eight to ten layers. **d, e** SEM images of CNTs array grown from graphene layer on Ni wire. **f** Cross-sectional SEM image of G/CNTs on nickel wire. **g, h** TEM images of G/CNTs with a seamless connection. **i** Cross-sectional SEM image of the tubular structure of G/CNTs hollow fiber. **j, k** SEM images of G/CNTs hollow fiber from side view. **l** SEM image of the connected area of graphene and CNTs in G/CNTs hollow fiber. **m, n** SEM images of G/CNTs/PANI hollow fiber from cross-sectional view. **o** SEM image of PANI nanowire array grown on the external surface of G/CNTs fiber. **p** High-resolution SEM image of PANI nanowire array inside the tubular of G/CNTs fiber

The hollow G/CNTs fibers were synthesized through a two-step chemical vapor deposition (CVD) method. As shown in Fig. 1, the detailed synthetic process is as follows. (1) Continuous few-layer graphene was grown around a nickel wire (step 1); then, a layer of catalyst (iron, Fe) and a buffer layer of Al_2_O_3_ were successively deposited around CVD-grown graphene of Ni wire by e-beam evaporation (step 2). (2) Vertically aligned CNTs were grown around graphene on Ni wire through a second CVD process (step 3). (3) The self-supported hollow G/CNTs fiber was achieved after etching off Ni wire substrate (step 4), and any other active materials (e.g., PANI) could be easily grown on both inner and outer surface of the obtained hollow G/CNTs fibers (step 5). Figure 2a, b shows SEM images of as-grown graphene around nickel wire, which has a typically wrinkled structure of layered materials. Graphene with eight to ten layers was further confirmed by TEM (Fig. 2c), which was mostly investigated in this work. Raman spectrum of graphene (Fig. S1) appears a peak of G-band at 1582 cm^−1^ and a peak of 2D-band at 2713 cm^−1^ without obvious D-band peak, indicating the as-grown graphene with high quality. From Fig. 2d–f, it can be clearly seen that aligned CNTs array radially grown around graphene/Ni fiber, which could also be confirmed from Raman spectrum (Fig. S1) appearing a typical D-band peak at 1332 cm^−1^. The height of CNTs array is approximately 16 μm with growth time of 4 min. TEM images (Fig. 2g, h) showed that the as-grown CNTs had walls from five to eight and external diameters from 8 to 10 nm. More interestingly, obvious junction between graphene and CNTs could be clearly observed, which revealed that CNTs were covalently grown from graphene layer (Fig. 2g, h). It is different from continuous graphene tube that will seriously shrink into graphene ribbon (Fig. S2), but the covalently connected G/CNTs maintained unique tubular structure (Fig. 2i) due to the support effect of outer grown CNTs even during removing of Ni substrate and drawing out from solution (Fig. S3). Meanwhile, the outer aligned CNTs collapsed and aggregated into compact format (Fig. 2j–l) due to solution effect, but CNTs always maintained highly ordered structure. The interface between graphene layer and roots of CNTs (Fig. 2l) could be clearly observed, which indicated that graphene and CNTs were toughly connected together, consistent with TEM results.

The unique self-supported tubular G/CNTs fiber could be used as an ideal platform to be functionalized with other active materials on both inner and outer surface of the tubes, achieving functional composite hollow fibers with high mass loading of active materials. In this regard, we deposited conducting polymer of PANI on both inner and outer surfaces of the self-supported hollow G/CNTs fibers through an in situ polymerization approach. The obtained G/CNTs/PANI composite fiber also had an interesting tubular structure without collapse (Fig. 2m), which could provide more effective surface area when it was used for fiber-shaped electrochemical relative devices (e.g., supercapacitor and DSSCs). From Fig. 2n–p, it can be clear seen that highly aligned PANI nanowires were grown on both inner and outer surfaces of the hollow G/CNTs fiber. Both EDX (energy-dispersive X-ray spectroscopy) mapping (Fig. S4) and Raman spectra (Fig. S5) can further confirm the successfully formed PANI. The weight contents of PANI in hollow G/CNTs/PANI composite fibers could be easily tuned from 72.6% to 89.8% with increasing the concentration of monomer aniline in precursor solution for polymerization from 6.7 to 20 mM. The specific weight (linear density) of G/CNTs/PANI hollow fibers fabricated by using precursor solution containing 10 mM aniline is about 12 μg cm^−1^, which is 6.3 times of G/CNTs hollow fibers (1.9 μg cm^−1^).

The electrical (Fig. S6) and mechanical (Fig. S7) properties of these fiber materials were also investigated. The electrical resistance of G/CNTs hollow fibers was about 236 Ω cm^−1^, slightly lower than that of bare graphene ribbon (293 Ω cm^−1^). The electrical resistance of G/CNTs/PANI hollow fibers obviously increased to 645 Ω cm^−1^ with deposition 72.6% of PANI because of the limited conductive property of polymer, and decreased to 368 Ω cm^−1^ with deposition 84.5% of PANI due to more CNTs bundled by the conductive polymer chains, and then increased to 521 Ω cm^−1^ with deposition 89.8% of PANI because of the aggregation of polymer. Meanwhile, the G/CNTs/PANI hollow fibers possessed higher tensile strength (12.4 MPa) than that of graphene ribbon (8.0 MPa) and G/CNTs (9.3 MPa) fibers. The relative low tensile strength of these fiber materials can be attributed to their hollow structure and low density. The hydrophilicity of G/CNTs and G/CNTs/PANI hollow fibers were evaluated because it will affect the wetting ability of aqueous electrolyte. The results (Fig. S8) showed that G/CNTs/PANI hollow fibers had better hydrophilicity than bare G/CNTs fibers, which greatly facilitates the infiltration of polymer electrolyte.

**Fig. 3:**
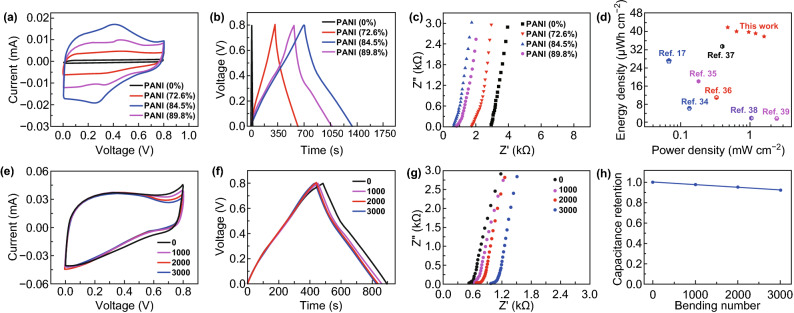
Electrochemical performance of supercapacitors based on G/CNTs and G/CNTs/PANI hollow fibers. **a–c** CV curves (scan rate of 10 mV s^−1^), GCD curves (current density of 0.6 mA cm^−2^) and Nyquist plots of the supercapacitors based on G/CNTs/PANI hollow fibers with different mass loading of PANI. **d** Comparison of energy density and power density of our supercapacitors with other reported fiber-shaped supercapacitors [17, 34–39]. **e–g** CV curves (scan rate of 10 mV s^−1^), GCD curves (current density of 1.0 mA cm^−2^) and Nyquist plots (10^−2^-10^5^ Hz) of supercapacitor under different bending cycles. **h** Capacitance retention of the supercapacitor during cyclic bending process

By using the obtained self-supported G/CNTs/PANI hollow fibers as both current collector and electrodes, fiber-shaped all-solid-state supercapacitors were developed with PVA-based gel electrolyte as separator simultaneously. Cyclic voltammogram (CV), galvanostatic charge–discharge (GCD) curves and electrochemical impedance spectroscopies (EIS) of supercapacitors were measured by using electrochemical working station. The areal specific capacitance of supercapacitors was calculated according to GCD curves by the equation of *C*_*s*_= *I*Δ*t*/*S*ΔV, where *I* is the discharge current, Δ*t* is the discharging time, *S* is the outer surface area of two-electrode supercapacitor and Δ*V* is the potential window. From CV curves shown in Fig. 3a, obvious redox peaks generated from conductive polymer of PANI could be observed, for the supercapacitors fabricated by using G/CNTs/PANI hollow fiber electrodes. All the GCD curves (Fig. 3b) of the fiber-shaped supercapacitors showed a typically symmetrical triangular structure, which indicated that the devices possessed an ideal capacitive behavior. According to the GCD curves, it can be calculated that the specific capacitance of supercapacitors increased from 225 to 472 mF cm^−2^ as the mass loading of PANI increased from 72.6% to 84.5%, then decreased to 365 mF cm^−2^ with mass loading of PANI further increasing to 89.5%. The highest specific capacitance of supercapacitors based on G/CNTs/PANI fibers with PANI loading of 84.5% was 472 mF cm^−2^ (corresponding to 232 F g^−1^, 2.75 mF cm^−1^, and 140.8 F cm^−3^, respectively), which was much higher than those (Fig. S9) of supercapacitors based on bare G/CNTs hollow fiber electrodes (3.1 mF cm^−2^) and bare graphene fiber electrodes (0.096 mF cm^−2^). The specific capacitance of our supercapacitors is comparative with other reported results (Fig. S10). The changes of specific capacitance of supercapacitors can be mainly contributed to the electrical resistance variation of the devices. As shown in Fig. 3c, the series resistance of supercapacitors decreased from 2.98 to 0.59 and 0.88 kΩ as the mass loading of PANI increased from 0 to 84.5% and 89.8%, because moderate polymer molecules can bundle CNTs together and promote the electrical conductivity of the composite fiber. More PANI loading will increase the resistance of the G/CNTs/PANI composite fibers, because of the limited conductivity of conducting polymer. The change of series resistance of supercapacitors based on G/CNTs/PANI hollow fibers with different loading of PANI was in well accordance with the variation of electrical resistance of G/CNTs/PANI hollow fiber electrodes (Fig. S6b). Besides, there was no obvious semicircle in high-frequency range (Fig. 3c), which indicated low charge transfer resistance of electrodes in devices. These supercapacitors also exhibited excellent rate performance (Fig. S11) and cycling performance (Fig. S12). To the best of our knowledge, the specific capacitance of our developed supercapacitor was among the highest range for fiber-shaped supercapacitors (Fig. 3d and Table S1) [17, 34–39]. Owing to the ultrahigh areal specific capacitance, our supercapacitors also possessed superior areal energy density and power density (42 μWh cm^−2^ at 0.48 mW cm^−2^ and 38 μWh cm^−2^ at 1.6 mW cm^−2^), compared with other reported fiber-shaped symmetrical supercapacitors (Fig. 3d). Benefiting from the high energy density of our fiber supercapacitors, a light-emitting diode (LED) can be lit up by five supercapacitors connected in series (Fig. S13). The newly developed supercapacitors not only had high electrochemical performance, but also showed excellent flexibility and mechanically bending stability. Their capacitance almost unchanged as the supercapacitor was bent with different angles (Fig. S14), suggesting good flexibility. As shown in Fig. 3e–h, the supercapacitor can maintain 92.2% of its original capacitance even after being bent for 3,000 times.

**Fig. 4:**
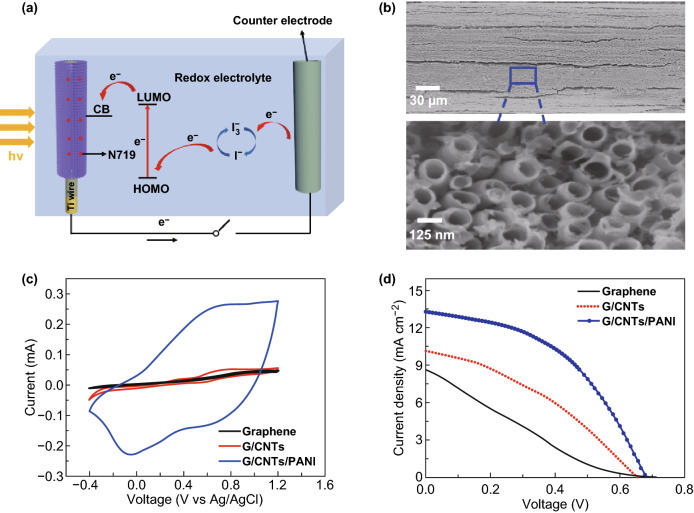
**a** Schematic working principle of fiber-shaped DSSC. **b** SEM image of a Ti wire grown with TiO_2_ nanotubes by electrochemical anodization for 2 h. **c** CV curves of graphene, G/CNTs and G/CNTs/PANI fibers in I^-^/I_3_^-^ electrolyte at scan rate of 50 mV s^−1^. **d** Current density–voltage (*J*–*V*) curves of DSSCs by using graphene, G/CNTs, and G/CNTs/PANI fibers as the counter electrodes

The G/CNTs/PANI hollow fibers can be used not only as electrodes for high-performance supercapacitors, but also as counter electrode replacing conventional platinum (Pt) wire for building fiber-shaped DSSCs (Fig. 4a). The working principle of the DSSC can be concluded as following. (1) The dye molecule of cis-diisothiocyanato bis(2,2’-bipyridyl-4,4’-dicarboxylato) ruthenium (II) bis(tetrabutylammonium) (denoted as N719) adsorbed on TiO_2_ nanotubes was excited under solar power, and generated electrons were then injected into the conduction band of TiO_2_. (2) The electrons afterward were transferred across external circuit and reached the counter electrode of G/CNTs/PANI fiber. (3) The I_3_^-^ in electrolyte captured electrons from counter electrode and was reduced to I^-^. (4) The excited dye molecule was reduced to ground state by receiving electron, which was produced from I^-^ oxidation to I_3_^-^ in redox electrolyte.

Figure 4b shows typical SEM image of the used working electrode where aligned TiO_2_ nanotubes were grown uniformly around Ti wire by an electrochemical anodization method. The electrochemical catalytic performance of the fiber electrodes toward the redox action of I^-^/I_3_^-^ redox couple was investigated. As shown in Fig. 4c, all the graphene, G/CNTs and G/CNTs/PANI fiber electrodes showed obvious catalytic activity for I^-^/I_3_^-^ redox couple. The peak current of the G/CNTs/PANI fibers was much higher than those of bare graphene and G/CNTs fibers, which suggested that G/CNTs/PANI fiber exhibited a better catalytic activity than graphene and G/CNTs fibers. The high catalytic activity of G/CNTs/PANI fiber can be ascribed to the rapid redox process of PANI and more active sites derived from the nanostructure of PANI nanowires. As a result, the fiber-shaped DSSC based on G/CNTs/PANI fiber electrode achieved a high power conversion efficiency of 4.20%, much higher than those of DSSCs by using bare Pt wire (3.41%), bare graphene (1.29%), and G/CNTs (2.54%) fiber electrodes (Fig. 4d and Fig. S15). The efficiency of our DSSC was comparable with other reported fiber-shaped DSSCs with Pt wire as the counter electrodes [40–42]. The high power conversion efficiency of our fiber-shaped DSSCs could be ascribed to the fast charge transfer of covalently connected G/CNTs and synergistic effect between PANI and nanocarbon materials. Furthermore, the as-fabricated DSSCs well maintained their high efficiency as they were bent at 0°, 45°, and 90° (Fig. S16), but cyclic mechanical stability of the DSSCs should be further optimized in the future.

**Fig. 5:**
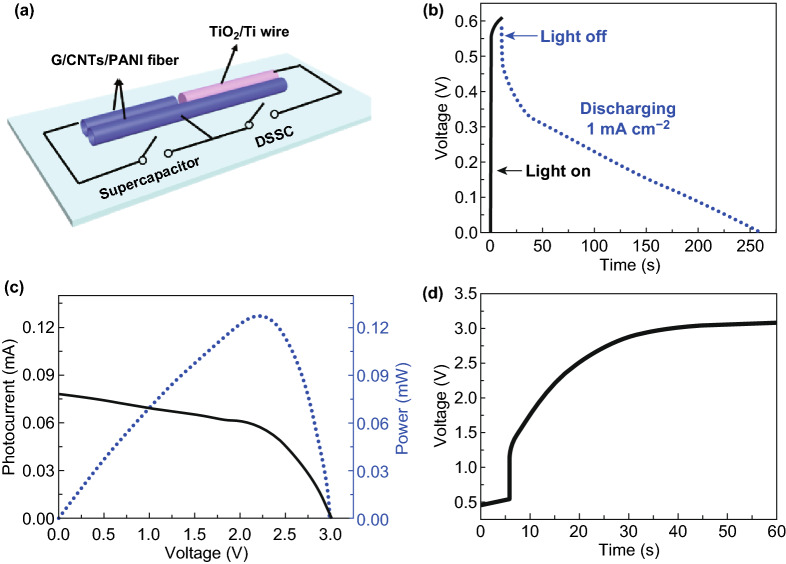
**a** Schematic illustration of integrated energy conversion and storage device with G/CNTs/PANI hollow fiber as the common electrode. **b** Dynamic voltage curves of photocharging and galvanostatic discharging processes of the integrated device. **c** Typical *J*–*V* curve (black curve) and voltage–power curve (blue curve) of five fiber-shaped DSSCs connected in series. **d** Solar-charging curve of five integrated devices connected in series

Fiber-shaped integrated device (Figs. 5a and S17) containing a DSSC and a supercapacitor was fabricated with one G/CNTs/PANI composite hollow fiber used as the common electrode. As shown in Fig. 5b, the supercapacitor part can be charged to 0.61 V, close to the open-circuit voltage (*V*_*oc*_) of DSSC, when the integrated device was exposed under irradiation of simulated solar light (one solar, power density of 100 mW cm^−2^) with the supercapacitor part connected with DSSC part. It means that simultaneous energy conversion and storage process was realized in the integrated device. Based on the power conversion efficiency of DSSC and energy storage efficiency of supercapacitor, it can be calculated that the total power conversion and storage efficiency of this integrated device reached 2.1%. The limited output voltage (0.6 V) of DSSC for charging supercapacitor was not enough for most electronics, which could be easily resolved by connecting several integrated devices in series. In this regard, five DSSCs connected in series could deliver an open-circuit voltage of 3.0 V and a photocurrent of 0.076 mA (Fig. 5c) under one solar light. As desired, fiber supercapacitors connected in series could be photocharged to 3.0 V (Fig. 5d), which was corresponding to the *V*_*oc*_ value of five DSSCs connected in series.

## Conclusions

In summary, we demonstrated a common fiber electrode of G/CNTs/PANI hollow fibers for high–performance integrated energy conversion and storage devices. Due to the support effect of CNTs directly grown from continuous graphene tube, the obtained G/CNTs hybrid fibers exhibited a unique self-supported hollow structure, which enabled them to being further functionalized with other active materials on both inner and outer surfaces of the tubes. To this end, conductive polymer of PANI that has been widely used for energy conversion and storage was decorated inner and outer of G/CNTs hollow fibers. The resultant G/CNTs/PANI composite hollow fibers can be used not only as electrodes to build supercapacitor with high specific capacitance of 472 mF cm^−2^, but also as efficient counter electrode for DSSC with a power conversion efficiency of 4.20%. The results proved that the developed G/CNTs/PANI composite hollow fibers could be used as a common electrode for both energy conversion and storage. As desired, the resultant integrated device of DSSC and supercapacitor can simultaneously store the electric energy generated by DSSC into the supercapacitor, and achieved a high total power conversion and storage efficiency of 2.1%. More importantly, the self-supported G/CNTs hollow fibers could be used as an ideal platform to be further functionalized with other active materials for application in various wearable electronics.

## Electronic supplementary material

Below is the link to the electronic supplementary material.

Supplementary material 1 (DOCX 3737 kb)
