# Supplementary material for: A Self-supported Graphene/Carbon Nanotube Hollow Fiber for Integrated Energy Conversion and Storage
Source: Nanomicro Lett. 2020 Feb 25;12:64. doi: 10.1007/s40820-020-0390-x (PMC7770695; doi:10.1007/s40820-020-0390-x)
Supplement: Supplementary file 1 — Supplementary material 1 (DOCX 3737 kb) [file 40820_2020_390_MOESM1_ESM.docx]

**Supporting Information**

**A Self-Supported Graphene/Carbon Nanotube Hollow Fiber for Integrated Energy Conversion and Storage**

Kai Liu, Zilin Chen, Tian Lv*, Yao Yao, Ning Li, Huili Li, Tao Chen*

Shanghai Key Lab of Chemical Assessment and Sustainability, School of Chemical Science and Engineering, and Institute of Advanced Study, Tongji University, Shanghai 200092, P. R. China. E-mail:18012@tongji.edu.cn (T. Lv), [tchen@tongji.edu.cn](mailto:tchen@tongji.edu.cn) (T. Chen).

**Fig. S1** Raman spectra of as-grown graphene (black line) and G/CNTs (red line) on Ni wires.


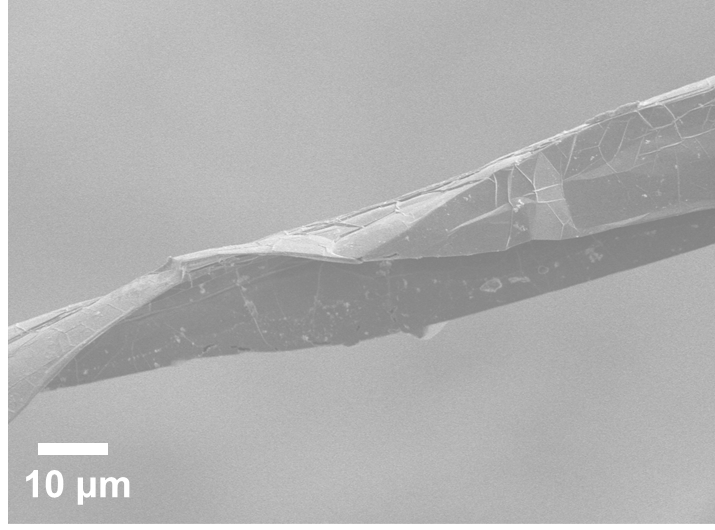


**Fig. S2** SEM image of graphene ribbon formed after shrinking of graphene tube.


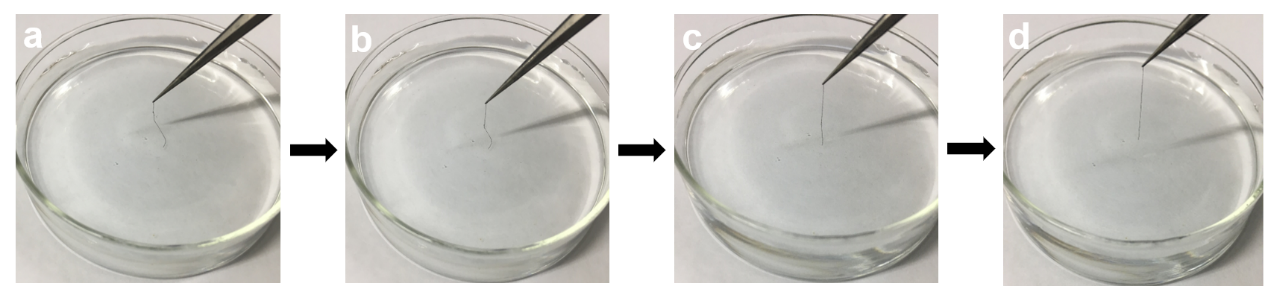


**Fig. S3** Digital photographs of an etched G/CNTs hollow fiber drawn out from water.


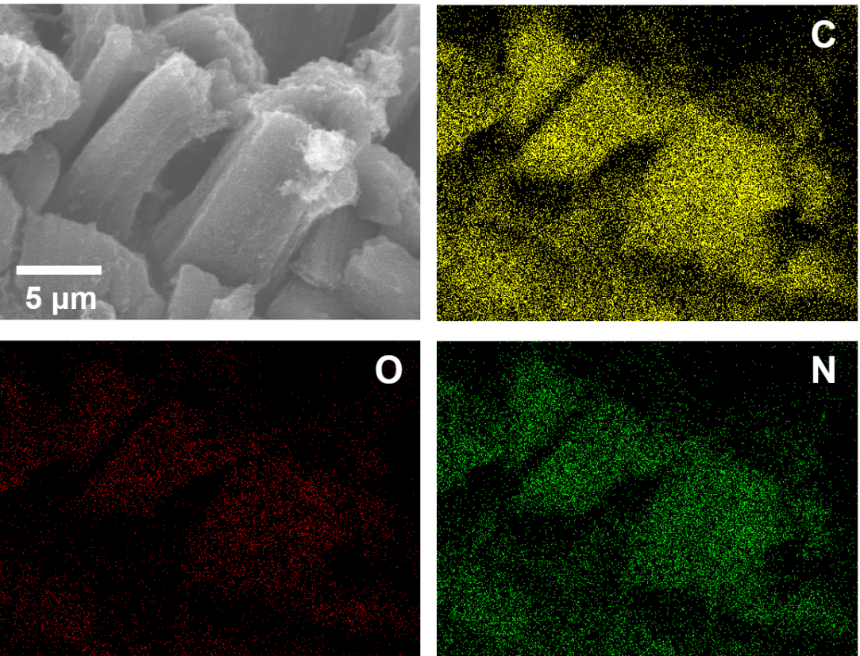


**Fig. S4** EDX element mapping of G/CNTs/PANI hollow fiber.

**Fig. S5** Raman spectra of G/CNTs/PANI hollow fibers synthesized by using precursor with different aniline contents.


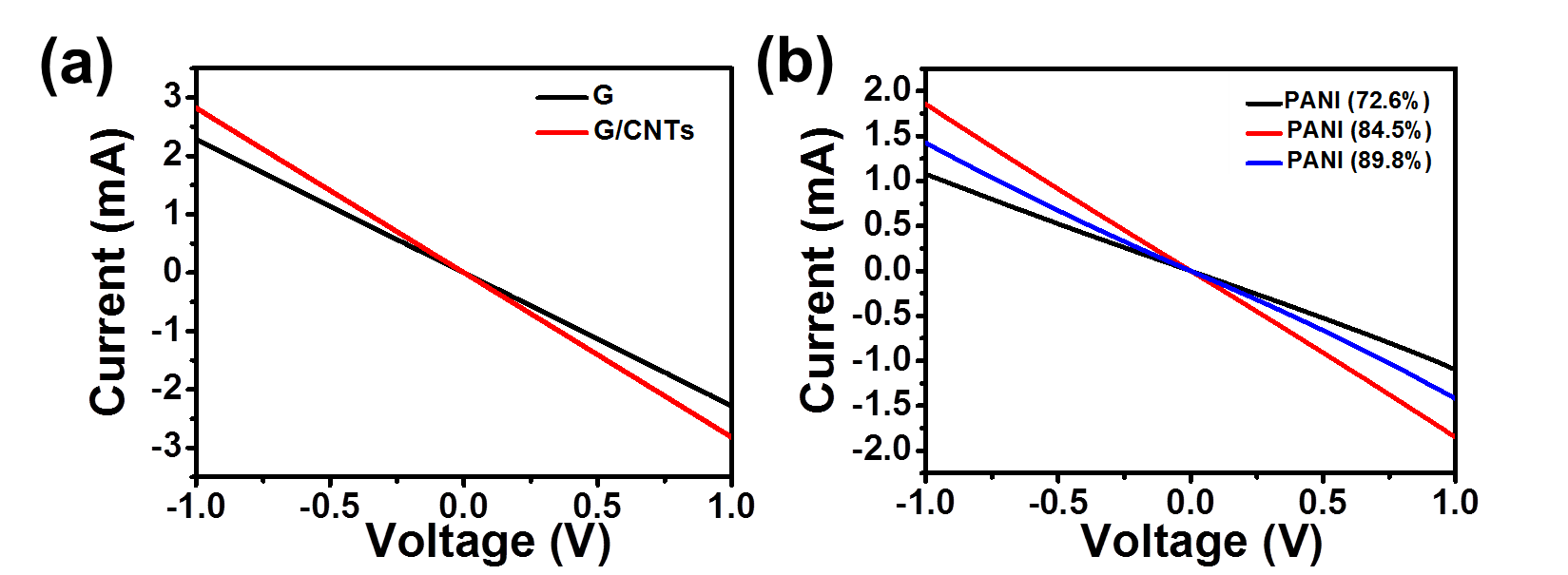


**Fig. S6** **a,b** I-V curves of graphene ribbon, G/CNTs fiber (a), and G/CNTs/PANI fibers with different mass loading of PANI (b).


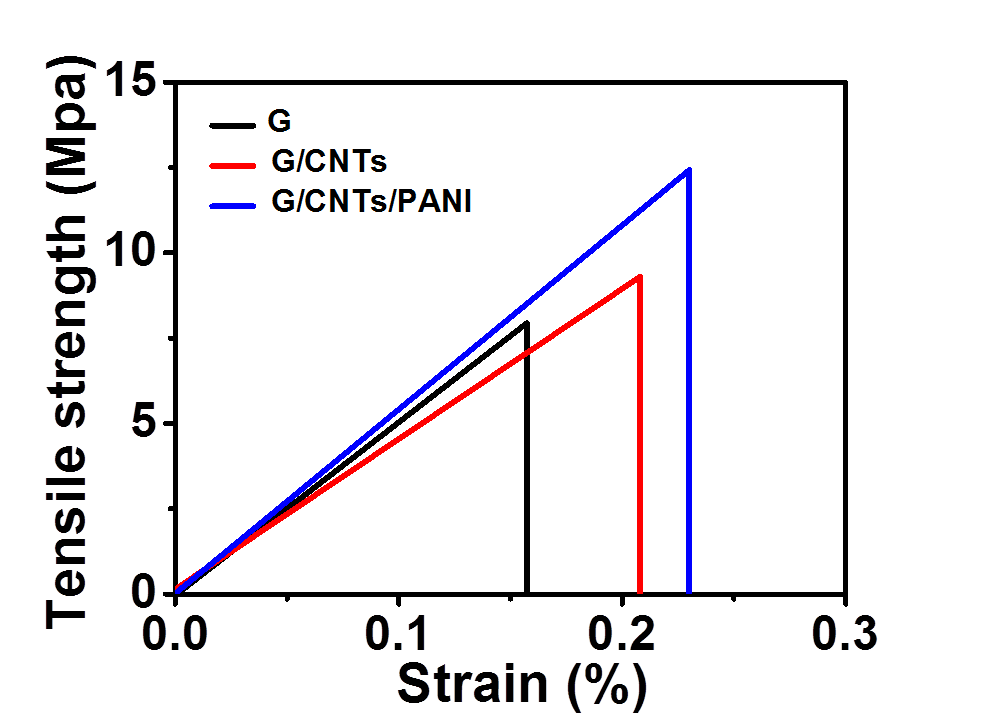


**Fig. S7** Tensile strength-strain curves of graphene ribbon, G/CNTs fiber and G/CNTs/PANI fiber.


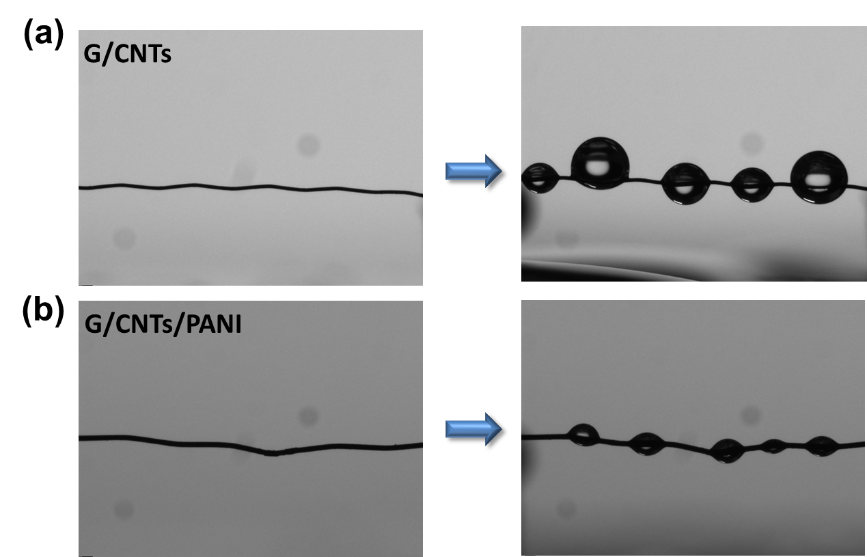


**Fig. S8** Photographs of water droplets on G/CNTs fiber (a) and G/CNTs/PANI electrodes (b).


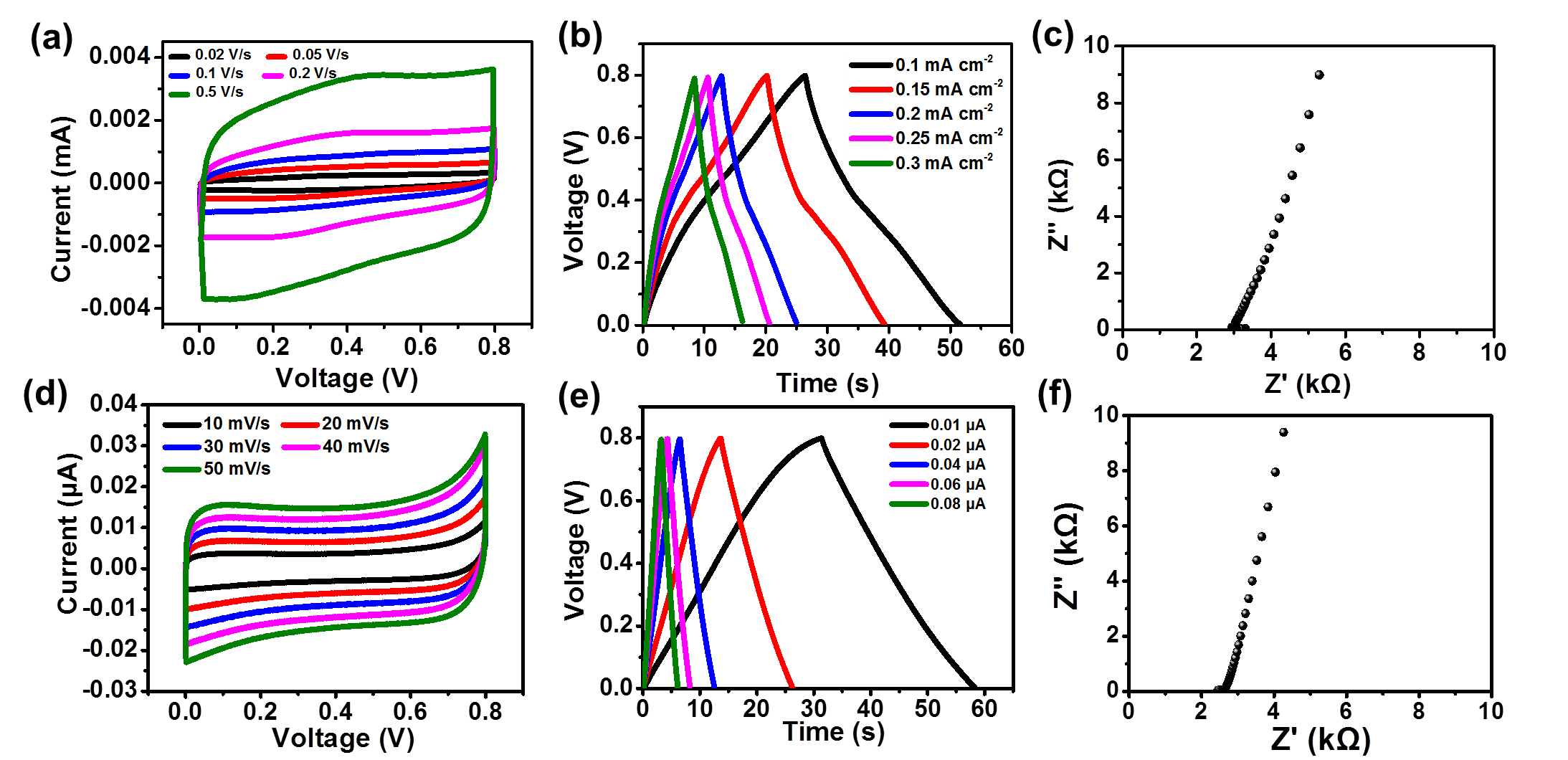


**Fig.** **S9** **a** CV curves of a fiber-shaped supercapacitor at scan rates range from 0.05 V s^-1^ to 0.5 V s^-1^. **b** GCD curves of the supercapacitor under different current density. **c** Nyquist plot of the supercapacitor within frequency from 10^-2^ to 10^5^ Hz. **d** CV curves of a fiber-shaped supercapacitor at scan rates range from 0.01 V s^-1^ to 0.05 V s^-1^. **e** GCD curves of the supercapacitor under different current densities. **f** Nyquist plot of the supercapacitor within frequency from 10^-2^ to 10^5^ Hz.


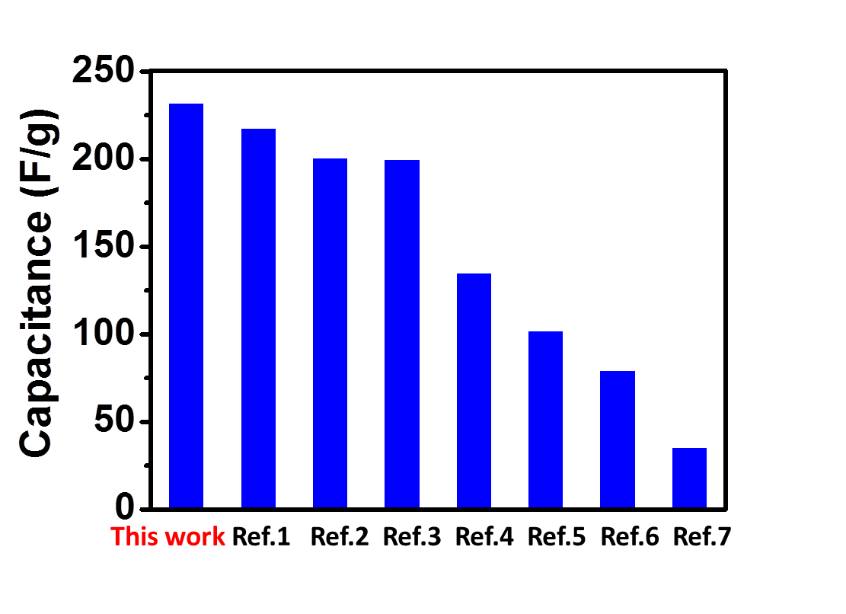


**Fig. S10** Comparison of gravimetric capacitance of our supercapacitors with other reported supercapacitors based on CNT/PANI electrodes [1-7].


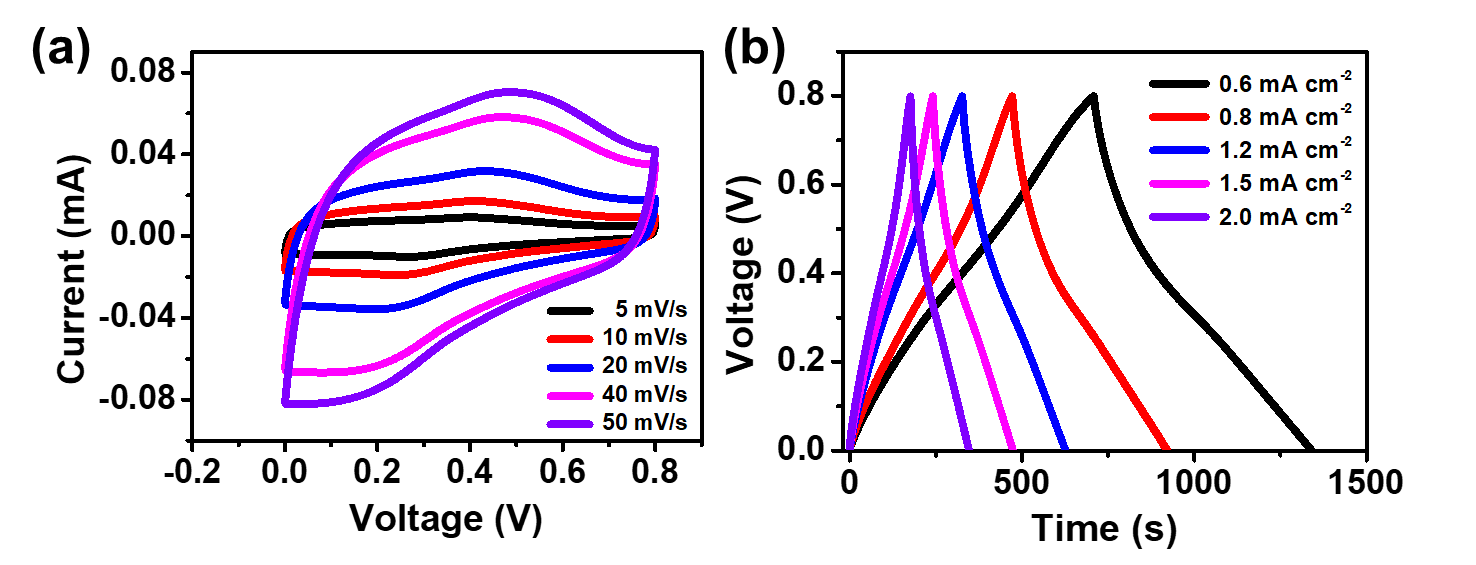


**Fig. S11** CV curves and GCD curves of fiber supercapacitor based on G/CNTs/PANI hollow fiber with PANI mass loading of 84.5%.


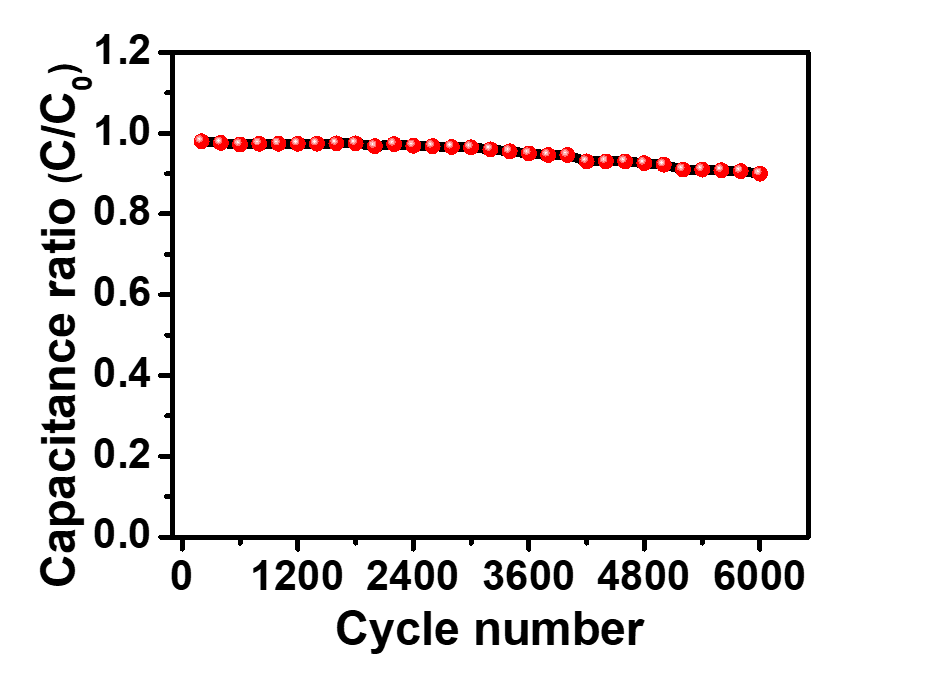


**Fig. S12** Cyclic performance of the fiber shaped supercapacitor.

**Table S1**. Comparison of our fiber shaped supercapacitor with other reported results.

| Device shape  (state of  electrolyte) | Configuration | Specific capacitance  (mF cm^-2^) | Reference |
| --- | --- | --- | --- |
| fiber (gel)  fiber (gel) | Graphene/CNTs/PANI  CNT/PANI array | **472**  37 | **This work**  [8] |
| fiber (gel) | CNT/PPy//CNT/MnO_2_ | 60.435 | [9] |
| fiber (gel) | CNT/graphene | 4.97 | [10] |
| fiber (gel) | rGO/PEDOT:PSS | 304.5 | [11] |
| fiber (gel) | rGO-Ni-polyester | 72.1 | [12] |
| fiber (liquid) | PANI/ Stainless steel | 41 | [13] |
| fiber (gel) | ppy/CNT | 69 | [14] |
| fiber (gel)  fiber (gel) | PEDOT@MnO_2_//  C@Fe_3_O_4_  RGO /CNT@CMC | 127  177 | [15]  [16] |


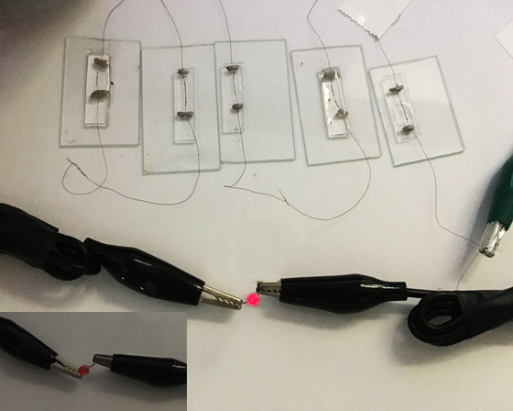


**Fig. S13** Digital photograph of five fiber supercapacitors connected in series to power a LED. The insert shows an LED without being powered.


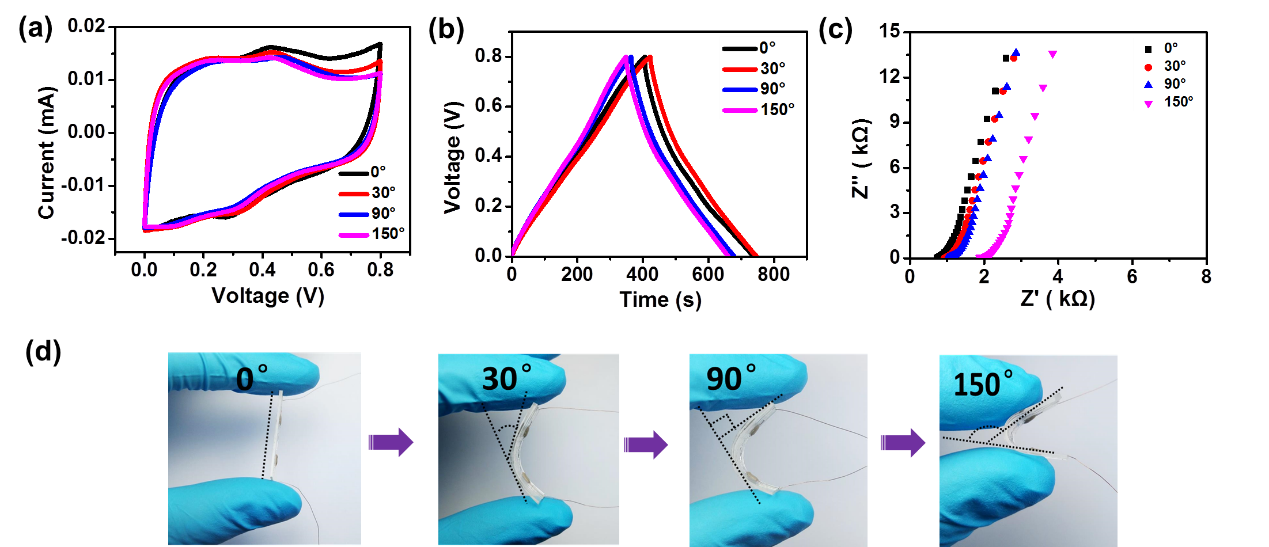


**Fig. S14** **a** CV curves (10 mV s^-1^), **b** GCD curves (1 mA cm^-2^) and **c** Nyquist curves (10^-2^-10^5^ Hz) of a fiber-shaped supercapacitor under different bending angles. **d** Digital photographs of a fiber-shaped supercapacitor under different bending states (0°, 30°, 90° and 150°).


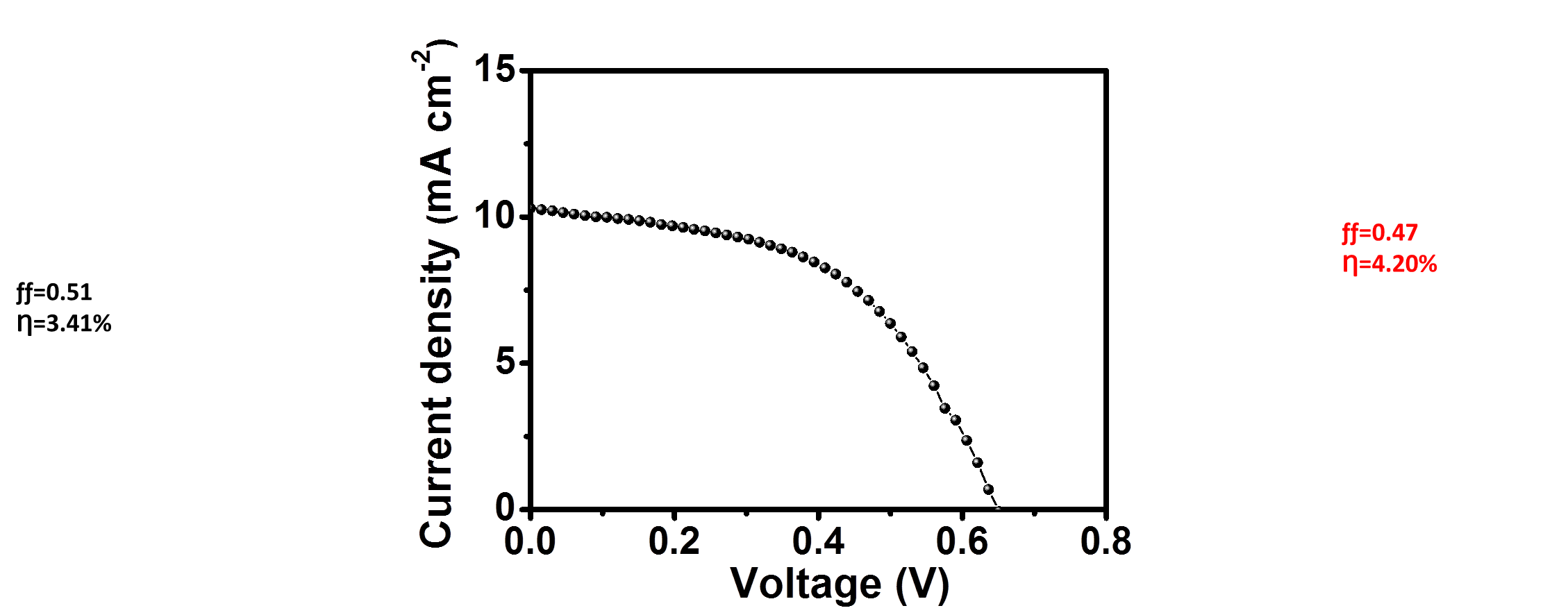


**Fig. S15** *J*-*V* curve of a DSSC by using Pt wire as the counter electrode.


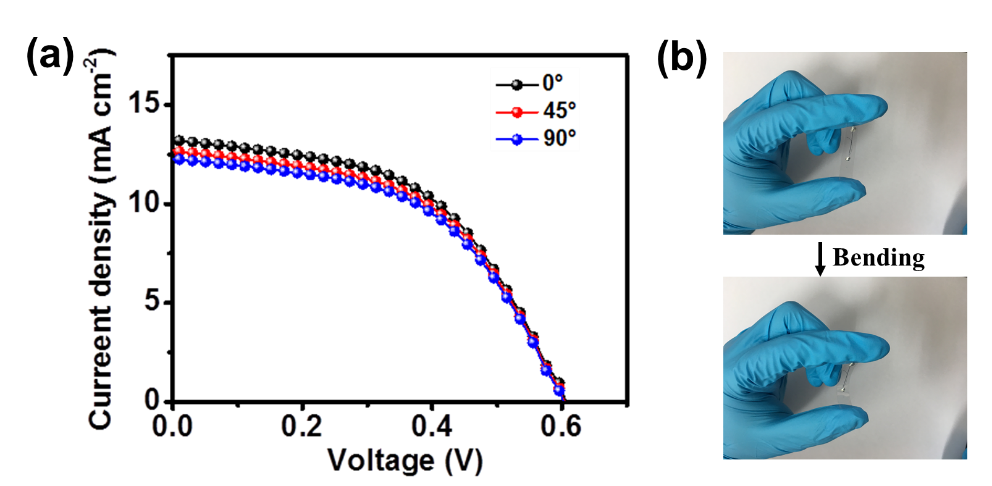


**Fig. S16** *J*-*V* curves and digital photographs of a fiber-shaped DSSC on an elastic substrate under different bending states.


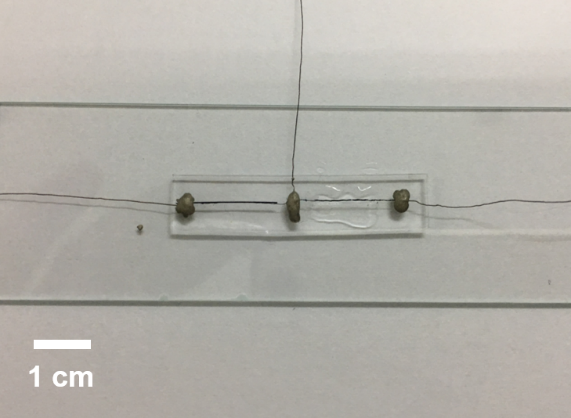


**Fig. S17** Digital photograph of an integrated energy conversion and storage device.

**Reference**

1. Y. Wu, Q. Wang, T. Li, D. Zhang, M. Miao, Fiber-shaped Supercapacitor and Electrocatalyst Containing of Multiple Carbon Nanotube Yarns and One Platinum Wire. Electrochim. Acta **245**, 69-78 (2017). <https://doi.org/10.1016/j.electacta.2017.05.117>
2. J. Li, W. Lu, Y. Yan, W. Chou, High performance solid-state flexible supercapacitor based on Fe_3_O_4_/carbon nanotube/polyaniline ternary films. J. Mater. Chem. A **5**, 11271-11277 (2017). <https://xs.scihub.ltd/10.1039/C7TA02008B>
3. J. Benson, I. Kovalenko, S. Boukhalfa, D. Lashmore, M. Sanghadasa, G. Yushin, Multifunctional CNT-Polymer Composites for Ultra-Tough Structural Supercapacitors and Desalination Devices. Adv. Mater. **25**, 6625-6632 (2013). <https://doi.org/10.1002/adma.201301317>
4. Q. Cheng, J. Tang, N. Shiny, Lu. Qin, Polyaniline modified graphene and carbon nanotube composite electrode for asymmetric supercapacitors of high energy density. J. Power Sources **241**, 423-428 (2013). <https://doi.org/10.1016/j.jpowsour.2013.04.105>
5. D. Potphode, P. Sivaraman, S. Mishra, M. Patri, Polyaniline/partially exfoliated multi-walled carbon nanotubes based nanocomposites for supercapacitors. Electrochim. Acta **155**, 402-410 (2015). <https://doi.org/10.1016/j.electacta.2014.12.126>
6. Z. Zhang, J. Deng, X. Li, Z. Yang, S. He, X. Chen, G. Guan, J. Ren, H. Peng, Superelastic Supercapacitors with High Performances during Stretching. Adv. Mater. **27**, 356-362 (2015). <https://doi.org/10.1002/adma.201404573>
7. S. He, L. Qiu, L. Wang, J. Cao, S. Xie, Q. Gao, Z. Zhang, J. Zhang, B. Wang, H. Peng, A three-dimensionally stretchable high performance supercapacitor. J. Mater. Chem. A **4**, 14968-14973 (2016). <https://doi.org/10.1039/C6TA05545A>
8. K. Wang, Q. Meng, Y. Zhang, Z. Wei, M. Miao, High-Performance Two-Ply Yarn Supercapacitors Based on Carbon Nanotubes and Polyaniline Nanowire Arrays. Adv. Mater. **25**, 1494-1498 (2013). https://doi.org/10.1002/adma.201204598

9. J. Yu, W. Lu, J.P. Smith, K.S. Booksh, L. Meng, Y. Huang, Q. Li, J.-H. Byun, Y. Oh, Y. Yan, T.-W. Chou, A High Performance Stretchable Asymmetric Fiber-Shaped Supercapacitor with a Core-Sheath Helical Structure. Adv. Energy Mater. **7**, 1066976 (2017). https://doi.org/10.1002/aenm.201600976

10. H. Sun, X. You, J. Deng, X. Chen, Z. Yang, J. Ren, H. Peng, Novel Graphene/Carbon Nanotube Composite Fibers for Efficient Wire-Shaped Miniature Energy Devices. Adv. Mater. **26**, 2868-2873 (2014). https://doi.org/10.1002/adma.201305188

11. G. Qu, J. Cheng, X. Li, D. Yuan, P. Chen, X. Chen, B. Wang, H. Peng, A Fiber Supercapacitor with High Energy Density Based on Hollow Graphene/Conducting Polymer Fiber Electrode. Adv. Mater. **28**, 3646-3652 (2016). https://doi.org/10.1002/adma.201600689

12. X. Pu, L. Li, M. Liu, C. Jiang, C. Du, Z. Zhao, W. Hu, Z.L. Wang, Wearable Self-Charging Power Textile Based on Flexible Yarn Supercapacitors and Fabric Nanogenerators. Adv. Mater. **28**, 98-105 (2016). https://doi.org/10.1002/adma.201504403

13. Y. Fu, H. Wu, S. Ye, X. Cai, X. Yu, S. Hou, H. Kafafy, D. Zou, Integrated power fiber for energy conversion and storage. Energy Environ. Sci. **6**, 805-812 (2013). https://doi.org/10.1039/C3EE23970E

14. J. Sun, Y. Huang, C. Fu, Z. Wang, Y. Huang, M. Zhu, C. Zhi, H. Hu, High-performance stretchable yarn supercapacitor based on PPy@CNTs@urethane elastic fiber core spun yarn. Nano Energy **27**, 230-237 (2016). https://doi.org/10.1016/j.nanoen.2016.07.008

15. J. Sun, Y. Huang, C. Fu, Y. Huang, M. Zhu, X. Tao, C. Zhi, H. Hu, A high performance fiber-shaped PEDOT@MnO_2_//C@Fe_3_O_4_ asymmetric supercapacitor for wearable electronics. J. Mater. Chem. A **4**, 14877-14883 (2016). https://doi.org/10.1039/C6TA05898A

16. L. Kou, T. Huang, B. Zheng, Y. Han, X. Zhao, K. Gopalsamy, H. Sun, C. Gao, Coaxial wet-spun yarn supercapacitors for high-energy density and safe wearable electronics. Nat. Commun. **5**, 3754 (2014). https://doi.org/10.1038/ncomms4754
